# The Use of Avatar Counseling for HIV/AIDS Health Education: The Examination of Self-Identity in Avatar Preferences

**DOI:** 10.2196/jmir.6740

**Published:** 2017-12-01

**Authors:** Shantrel Canidate, Mark Hart

**Affiliations:** ^1^ Social and Behavioral Sciences Program College of Public Health and Health Professions University of Florida Gainesville, FL United States

**Keywords:** technology, distance education, learning, avatars

## Abstract

**Background:**

The number of adults using the Internet to obtain health information is on the rise. An estimated 66% of the adults reportedly use the Internet to obtain health information related to a specific disease (ie, human immunodeficiency virus and acquired immunodeficiency syndrome, HIV/AIDS). Previous research has demonstrated that health information seekers use the Internet to seek answers to stigma-laden questions from health avatars.

**Objective:**

The objective of this study was to identify patterns in the choice of avatar among health information seekers (patients or public health workers) using the Internet to obtain HIV/AIDS information and to describe the demographic characteristics (age, gender, and ethnicity) of health information seekers to determine whether they preferred an avatar that was similar to their own gender and ethnicity.

**Methods:**

The Rural South Public Health Training Center (RSPHTC) partnered with the New York State Department of Health to create the HIV/AIDS Avatar project. The avatar project was created to serve as an educational resource for public health workers by providing relevant and accurate information about HIV/AIDS. First, the user was instructed to choose one of the 8 avatars that voiced responses to 100 common questions and answers about HIV/AIDS. Next, the website gave users the option to complete a brief 3-question demographic survey. Finally, the demographic characteristics of each user were compared with the chosen avatar to determine whether they preferred an avatar that was similar to their own gender and ethnicity.

**Results:**

The avatar project website was loaded with 800 videos that included the answers to the top 100 questions about HIV/AIDS voiced by 8 avatars. A total of 1119 Web-based health information seekers completed the demographic survey upon accessing the website. Of these, 55.14% (617/1119) users were female. A total of 49.96% (559/1119) users were aged between 30 and 49 years. The ethnicity of the user and the avatar was found to have the strongest connection. All the users choose the female avatar matching their own ethnicity, followed by the male avatar. Additionally, the white female avatar was chosen the most by all users regardless of the age group or gender.

**Conclusions:**

Web-based health information seekers using the Internet to access medical research information may feel more comfortable receiving the answers to HIV stigma-laden questions from avatars, rather than receiving information directly from a health care provider. Additionally, providers seeking to utilize avatars to deliver interventions in health care settings may benefit from offering individuals choices in how they receive health information. Having the ability to choose whom you seek information from may lead to an increase in knowledge and awareness and could motivate HIV-positive individuals to seek care.

## Introduction

Human immunodeficiency virus (HIV) infection is a major global public health problem. Currently, an estimated 1 million people in the United States are living with HIV, and 39 million people are living with the virus worldwide [1]. In the United States, nearly 240 new cases of HIV occur every hour, and an estimated 50,000 people become infected with HIV each year [2]. Despite global actions being taken toward reducing stigma, ignorance, and discrimination, ensuring that individuals have access to the latest and most accurate information about the risks associated with HIV infection is important [3].

Now, more than ever, the Internet has become a primary source for gathering health information among adults. As of 2013, approximately 72% of the adults used the Internet to access Web-based health information. An additional 66% of the adults reportedly used the Internet to access information pertaining to a specific disease (ie, human immunodeficiency virus infection and acquired immunodeficiency syndrome, HIV/AIDS) [4]. Due to the recent growth of Internet-based access to medical and research information, health information seekers (ie, patients or public health workers) are beginning to seek answers to stigma-laden questions from avatars, as opposed to asking a health care provider. In Hinduism, an avatar or *avatara* is a Sanskrit word meaning *descent* and refers to the incarnation (bodily manifestation) of an immortal being [5]. In computing, the definition of an *avatar* varies. Miroslaw Filiciak defines an avatar as the “user’s representative in the virtual universe,” whereas Chris Crawford’s definition defines them as “virtual constructs controlled by human players and function as a means of interacting with other characters.” Moreover, Anya Wood defines avatars as the “computer-generated characters that are used to represent a human” [5-7]. Although avatars can be used to represent oneself, the concept of identity has mostly been related to computing and not health [8-10].

Identity or *sameness* is a driving force in determining who we are as a person. Although several studies have examined identity and avatars in computing [8-10], little research exists on how users select an avatar’s gender and ethnicity when searching for medical information. With the increase in the use of health avatars by users to access medical information, researchers are trying to answer the question regarding the importance of the concept of identity. Specifically, researchers are seeking to examine whether participation and user satisfaction will increase if more resources are used to create avatar choices of all races and genders among other populations who might be using avatars. Specifically to HIV, avatar-based public health trainings place the control parameters of the HIV epidemic in the hands of each individual by providing tools that are easy to understand and use [11]. The purpose of this exploratory study was to identify patterns in choice of avatar among health information seekers (patients or public health workers) using the Internet to obtain HIV/AIDS information and to describe the demographic characteristics (age, gender, and ethnicity) of health information seekers to determine whether they preferred an avatar that was similar to their own gender and ethnicity.

## Methods

### Overview of the Rural South Public Health Training Center

The Rural South Public Health Training Center (RSPHTC) served medically underserved counties in Florida, particularly in the northern rural regions of the state. The training center provided competency-based training programs for public health workers, enhanced public services, and improved community access to services. Thus, Web-based health information seekers pursuing continuing education credits were able to access a website where the answers to *The Most Common 100 Questions and Answers about HIV/AIDS* were voiced by an avatar.

### The HIV/AIDS Avatar Project

#### Avatar and Website Creation

**Figure 1:**
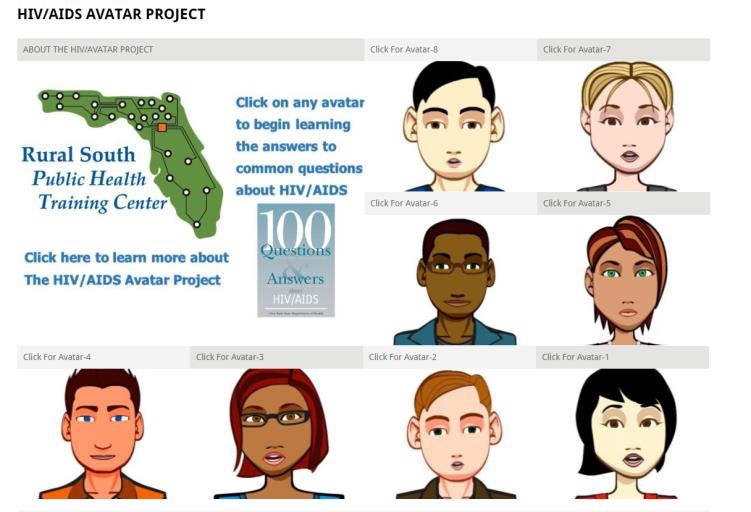
This is a screen capture of five of the eight avatars that users could choose to hear responses to the 100 common questions about HIV/AIDS.

The RSPHTC partnered with the New York State Department of Health to create the HIV/AIDS Avatar project. The avatar project was designed to provide relevant and factual information for patients and to serve as a tool for public health workers. Voki.com, a creative and easy-to-use educational tool, was used to create the avatars. A total of 8 avatars (4 male and 4 female) were created using the simple interface’s default settings (ie, character style, customization, voice, and background) (Figure 1). Character style included selecting classic avatars to represent the top 4 racial/ethnic groups and subgroups in the United States according to the information from the US Census Bureau. The top 4 groups were as follows: white, African American, Hispanic, and Asian American. Next, customization was used to select a theme of neutral colors, casual clothes, etc, of each avatar. Then, using text-to-speech, voices for genders and ethnicities were added to each avatar. The final step involved choosing a background image for each of the avatars and publishing the avatars to the website. Once the avatars were placed on the website, they were randomly moved around so that *proximity* did not alter findings. Furthermore, the avatars were displayed on the same page in a linear display around the logo.

#### Participant Recruitment

The research team employed various recruitment strategies. Participants were recruited through emails, newsletters, and snowball sampling. Information pertaining to the HIV/AIDS Avatar project website was sent via emails and newsletters to thousands of public health workers across the state of Florida. Information was also forwarded to participants at the University of Florida Health and the Suwannee River Area Health Education Center. The research team also placed advertisements on the RSPHTC website and on YouTube.

**Table 1:** Demographic questionnaire.

Item	Question	Response
Age	What is your age?	18-29 years old
		30-49 years old
		50-64 years old
		65 years and older
Gender	What is your gender?	Male (M)
		Female (F)
Ethnicity origin (or race)	Please specify your ethnicity.	Black or African American
		Asian
		White
		Latino

#### Data Collection

Upon accessing the project website, the user could select one of the 8 avatars to deliver HIV/AIDS information. Next, after selecting an avatar, the user was given the option to complete a demographic questionnaire (see Table 1). If the users chose to not provide the demographic information, they were redirected to the website where they could access the content directly. The users who chose to complete the questionnaire were asked to report their age, gender, and ethnicity origin (or race). The users’ responses were used by the research team for statistical tracking of the chosen avatar in relationship to the demographic information provided by the user. Furthermore, no self-identifiers or Internet Protocol addresses were collected. All the users were given the option of choosing the information they wanted, with questions grouped by category (ie, basic information and incidence/trends, transmission, testing, risk reduction, and diagnosis and treatment). The project website was also equipped with Google Analytics (Google), which was used to determine the platform the users were using (ie, iPhone operating system and Android), users’ accessibility to the website (ie, laptop or cell phone), and the location of the users (ie, region, city, and/or state). This information was not connected to the demographic survey in any way.

#### Data Analysis

The research team used the backend analytics, embedded in the HIV/Avatar website, to access the data. Raw counts were used to stratify the users’ age, gender, and ethnicity (or race) linked with the avatar chosen by the user to receive information on HIV/AIDS. Next, for each avatar, a corresponding count was associated with the response to the demographic questionnaire variable (ie, age, gender, and ethnicity) that was voluntarily completed by the user before accessing the website’s content. The raw counts were analyzed and converted to percentages of those chosen. Over 1000 users responded to the demographic questionnaire. Although this was an initial look at the pattern of choice of avatar in relation to the users’ responses to the demographic variables, the data remained focused on these 3 variables singularly. Further data collection and analysis, however, could lead to a larger group of respondents, which would further enhance the data to cross-reference the variables (ie, which avatar a *female* / *18-29* / *Latino* selected).

## Results

The study included 1119 health information seekers who completed the demographic questionnaire. The questionnaire was used to collect information on participants’ age, gender, and ethnicity. The characteristics of the users are presented in Table 2. Of the 1119 users, 325 users (29.04%) were aged between 18 and 29 years, 559 users (49.96%) were aged between 30 and 49 years, 209 (18.68%) were aged between 50 and 64 years, and 26 users (2.32%) were in the age group of 65 years and older. In addition, 502 (44.86%) users were men, and 617 (55.14%) were women. Among the users who completed the demographic questionnaire, 224 (20.02%) were black or African American, 522 (46.65%) were white, 287 (25.65%) were Latino, and 86 (7.68%) were Asian.

The characteristics of the users in relation to the chosen avatar are shown in Table 3.

Among the 325 users in the age group of 18 to 29 years, 74 (22.8%) chose the white female avatar, followed by 57 (17.5%) who chose the black male avatar. Additionally, 112 (20.0%) users in the age group of 30 to 49 years chose the white female avatar, whereas 111 (19.9%) chose the black female avatar. Approximately 52 (24.9%) users in the age group of 50 to 64 years chose the white female avatar, and 34 (16.3%) chose the white male avatar. Among those aged 65 years and older, 11 (42%) chose the white female avatar, followed by 4 (15%) who chose the white male avatar.

An estimated 108 (21.5%) male users chose the white female avatar, followed by 97 users (19.3%) who chose the black male avatar. On the contrary, 141 (22.9%) female users chose the white female avatar, and 111 (18.0%) chose the black female avatar.

**Table 2:** Characteristics of Web-based health information seekers (N=1119).

Demographic characteristics		n (%)
**Age group (years)**		
	18-29	325 (29.04)
	30-49	559 (49.96)
	50-64	209 (18.68)
	65 and older	26 (2.32)
**Gender**		
	Men	502 (44.86)
	Women	617 (55.14)
**Race/ethnicity**		
	Black or African American	224 (20.02)
	Asian	86 (7.68)
	White	522 (46.65)
	Latino	287 (25.65)

**Table 3:** Demographics of the user and the selected avatar.

User demographics		Avatar demographics							
		Black		Asian		White		Latino	
		Male, n (%)	Female, n (%)	Male, n (%)	Female, n (%)	Male, n (%)	Female, n (%)	Male, n (%)	Female, n (%)
**Age group (years)**									
	18-29	57 (17.5)	52 (16.0)	15 (4.6)	32 (9.8)	37 (11.4)	74 (22.8)	32 (9.8)	26 (8.0)
	30-49	66 (11.8)	111 (19.9)	44 (7.9)	46 (8.2)	68 (12.2)	112 (20.0)	34 (6.1)	78 (14.0)
	50-64	24 (11.5)	23 (11.0)	17 (8.0)	17 (8.0)	34 (16.3)	52 (24.9)	15 (7.2)	27 (12.9)
	65 and older	2 (8)	3 (11.5)	0 (0)	1 (4)	4 (15)	11 (42)	3 (11.5)	2 (8)
**Gender**									
	Male	97 (19.3)	78 (15.5)	18 (3.6)	37 (7.4)	96 (19.1)	108 (21.5)	34 (6.8)	34 (6.8)
	Female	52 (8.4)	111 (18.0)	58 (9.4)	59 (9.6)	47 (7.6)	141 (22.9)	50 (8.1)	99 (16.0)
**Ethnicity**									
	Black	65 (29.0)	82 (36.6)	8 (3.6)	5 (2.2)	15 (6.7)	31 (13.8)	4 (1.8)	14 (6.3)
	Asian	10 (11.6)	5 (5.8)	12 (14.0)	25 (29.1)	11 (12.8)	10 (11.6)	5 (5.8)	8 (9.3)
	White	41 (7.9)	63 (12.1)	31 (5.9)	39 (7.5)	98 (18.8)	176 (33.7)	25 (4.8)	49 (9.4)
	Latino	33 (11.4)	39 (13.6)	25 (8.7)	27 (9.4)	19 (6.6)	32 (11.1)	50 (17.4)	62 (21.6)

Among black users who completed the demographic questionnaire, 82 (36.6%) chose the black female avatar, and 65 (29.0%) chose the black male avatar. Twenty-five (29.1%) Asian users chose the Asian female avatar, followed by 12 (14.0%) who chose the Asian male avatar. Moreover, 176 (33.7%) white users chose the white female avatar, whereas 98 (18.8%) chose the white male avatar. Additionally, in Latino users, 62 (21.6%) chose the Latino female avatar, followed by 50 (17.4%) who chose the Latino male avatar.

## Discussion

### Principal Findings

Our exploratory study sought to identify the patterns in the choice of avatar among health information seekers (patients or public health workers) using the Internet to obtain HIV/AIDS information and to describe the demographic characteristics (age, gender, and ethnicity) of health information seekers to determine whether they preferred an avatar that was similar to their own gender and ethnicity. A total of 1119 users completed the voluntary demographic questionnaire. As highlighted in the results, 559 (49.96%) of the users were aged between 30 and 49 years, followed by 325 (29.04%) who were in the age group of 18 to 29 years. Furthermore, 235 (21.00%) of the users were aged 50 years and older, despite many older individuals being sexually active, including those who are living with HIV and may have many of the HIV risk factors as younger individuals [12]. Among gender groups, 115 more female users completed the demographic questionnaire as compared with the male users. Among the male and female users, the white female avatar was chosen the most. Thus, female avatars tend to be highly regarded among many female gamers. Previous research has demonstrated that women who play video games have concerns about the availability and physique of a female avatar [13]; whether or not this holds true for Web-based health information seekers is unknown. Furthermore, the ethnicity of the user and the ethnicity of the avatar were found to have the strongest connection. The black, Asian, white, and Latino users each chose the male and female avatar representing their own ethnicity. Regardless of the age and gender, majority of users chose the white female avatar, followed by either the white male avatar or the black male or female avatar. Moreover, black participants *exposed to the low-diversity representation of Second Life* were shown to create more white-looking avatars as opposed to black participants *exposed to the high-diversity representation* [14].

This study has significant implication for health care practitioners. Although the use of avatar technology in the medical field is relatively new, health avatars could provide health care practitioners with a new set of tools allowing for better tracking of patients while boosting engagement in care, improving treatment adherence, and decreasing costs [15]. Previous research shows that individuals view avatars just as they view those around them. Thus, if more human-looking avatars are available, users may be willing to open up to them [15]. As denoted by the study findings, creating multiple ethnic avatars may be best, given that the study users were more likely to pick avatars of their own ethnicity. However, if you could only create one avatar for a project, the white female avatar is the most preferred (according to study findings). Among individuals who identify with their chosen avatar, it is hoped that they would be empowered to adopt healthier behavioral choices as a result of the information they receive about the virus through the virtual world [11]. Eventually, using avatars to deliver health information may become the new norm, given that they are readily available, user-friendly, and capable of providing specialty care at the tertiary level [15].

### Limitations

We noted several potential limitations as context for interpreting our findings. Due to the self-reported nature of the questionnaire, we were unable to determine whether the information reported by the users was accurate. Also, assuming that the users who accessed the project website were either patients or public health care workers may cause some to question the external validity of our findings. Due to the anonymity of the users who completed the demographic questionnaire, we were also unable to determine whether the user was in fact a patient or public health worker. Among the users who accessed the website, we are unable to determine their reasons for seeking the health information. Currently, we cannot determine whether the users were looking for answers to stigma-laden questions because they have been asked these questions before or for self-educating purposes. Although minority individuals with low socioeconomic status (SES) or men who have sex with men have a higher risk of contracting HIV infection, this study did not capture information on these groups. Pursuing this further, the demographic questionnaire asked the users to self-report their age, gender, and ethnicity; however, once the user accessed the website, there was information pertaining to HIV/AIDS that was applicable for everyone. Thus, future research could allow for a better-designed questionnaire that could ask broader questions pertaining to the users’ SES, sexual orientation, and marital status. Furthermore, in this exploratory study, we did not account for users who did not identify as either male or female or as both. As completing the demographic questionnaire was optional, we were unable to determine whether users chose not to complete the survey because they did not identify as either male or female, or they identified as both male and female. Future research should allow the user to write in their gender preference if they do not identify as male or female. Lastly, there was no variation in the age of the 8 avatars that were used in this study. As a result, we were unable to determine whether there was a relationship between the age of the information seeker and the age of the avatar. Future studies seeking to examine patterns of choice in avatars among health information seekers may benefit from creating avatars that vary in gender, ethnicity, and age.

### Conclusions

In summary, HIV/AIDS is a disease that continues to affect millions of individuals around the world. Although several social, economic, and demographic factors increase an individual’s risk of contracting HIV, ensuring that individuals have access to the most accurate information is imperative. With an increase in the number of people who are using the Internet to access health information, research demonstrates that many individuals are turning to avatars to seek answers to stigma-laden medical questions. Therefore, health care providers are encouraged to offer multiple avatars, given that people will pick avatars of their own ethnicity. Study findings showed that the white female was the most preferred avatar. Additionally, avatar-based interventions can be used to allow individuals to visualize possible self by providing more *salient and concrete* information about the future, which can motivate individuals to change existing behavior for the sake of future benefits.

## Abbreviations

AIDS: acquired immunodeficiency syndrome
HIV: human immunodeficiency virus
RSPHTC: Rural South Public Health Training Center
SES: socioeconomic status
